# Proximity-Dependent Biotinylation and Identification of Flagellar Proteins in Trypanosoma cruzi

**DOI:** 10.1128/msphere.00088-23

**Published:** 2023-04-05

**Authors:** Madalyn M. Won, Aaron Baublis, Barbara A. Burleigh

**Affiliations:** a Department of Immunology and Infectious Diseases, Harvard T. H. Chan School of Public Health, Boston, Massachusetts, USA; b Harvard Chan Advanced Multi-omics Platform, Harvard T. H. Chan School of Public Health, Boston, Massachusetts, USA; University at Buffalo

**Keywords:** *Trypanosoma cruzi*, TurboID, amastigote, flagellum, proteomics, proximity labeling

## Abstract

The flagellated kinetoplastid protozoan and causative agent of human Chagas disease, Trypanosoma cruzi, inhabits both invertebrate and mammalian hosts over the course of its complex life cycle. In these disparate environments, T. cruzi uses its single flagellum to propel motile life stages and, in some instances, to establish intimate contact with the host. Beyond its role in motility, the functional capabilities of the T. cruzi flagellum have not been defined. Moreover, the lack of proteomic information for this organelle, in any parasite life stage, has limited functional investigation. In this study, we employed a proximity-dependent biotinylation approach based on the differential targeting of the biotin ligase TurboID to the flagellum or cytosol in replicative stages of T. cruzi to identify proteins that are enriched in the flagellum by mass spectrometry. Proteomic analysis of the resulting biotinylated protein fractions yielded 218 candidate flagellar proteins in T. cruzi epimastigotes (insect stage) and 99 proteins in intracellular amastigotes (mammalian stage). Forty of these enriched flagellar proteins were common to both parasite life stages and included orthologs of known flagellar proteins in other trypanosomatid species, proteins specific to the T. cruzi lineage and hypothetical proteins. With the validation of flagellar localization for several of the identified candidates, our results demonstrate that TurboID-based proximity proteomics is an effective tool for probing subcellular compartments in T. cruzi. The proteomic data sets generated in this work offer a valuable resource to facilitate functional investigation of the understudied T. cruzi flagellum.

**IMPORTANCE** Trypanosoma cruzi is a protozoan parasite that causes Chagas disease, which causes substantial morbidity and mortality in South and Central America. Throughout its life cycle, T. cruzi interacts with insect and mammalian hosts via its single flagellum, establishing intimate contact with host membranes. Currently, few flagellar proteins have been identified in T. cruzi that could provide insight into the mechanisms involved in mediating physical and biochemical interactions with the host. Here, we set out to identify flagellar proteins in the main replicative stages of T. cruzi using a proximity-labeling approach coupled with mass spectrometry. The >200 candidate flagellar proteins identified represent the first large-scale identification of candidate flagellar proteins in T. cruzi with preliminary validation. These data offer new avenues to investigate the biology of T. cruzi-host interactions, a promising area for development of new strategies aimed at the control of this pathogen.

## INTRODUCTION

Trypanosoma cruzi is the uniflagellate protozoan parasite that causes Chagas disease, a chronic disease with severe outcomes including cardiomyopathies and gastrointestinal motility disorders (1, 2). T. cruzi has a complex life cycle that involves both invertebrate and mammalian hosts, in which the parasite undergoes marked developmental changes and alternates between actively dividing (epimastigote and amastigote forms in insect and mammalian hosts, respectively) and nondividing trypomastigote forms in both hosts (see the life cycle schematic in Fig. S1 in the supplemental material). In mammals, infection is initiated by motile trypomastigotes that actively invade host cells before converting to the nonmotile amastigote stage that replicates in the host cytoplasm. Intracellular T. cruzi amastigotes begin to replicate ~24 h postinfection (hpi) and undergo several rounds of cell division before converting back to trypomastigotes, which eventually rupture the host cell membrane (between ~90 and 120 hpi) to allow dissemination of the parasite and infection of new tissue sites. Once T. cruzi infection is established in mammalian hosts, parasites typically persist at low levels for the life of the host, giving rise to chronic infections that can trigger inflammation and pathology.

10.1128/msphere.00088-23.1 FIG S1 Schematic of the T. cruzi life cycle. The insect stage (epimastigote) is propagated axenically in liquid culture and gives rise to the infectious metacyclic trypomastigote. Trypomastigotes, whether derived from epimastigotes or as the end product of a single lytic cycle in a mammalian cell, are motile, nondividing forms of the parasite that actively invade a mammalian host cell. Inside a host cell, the trypomastigote transforms into the replicative intracellular amastigote stage by 18 h postinfection (hpi). Amastigotes undergo several rounds of proliferation, dividing by binary fission (between ~24 and 90 hpi), before they stop dividing and differentiate into trypomastigotes, which eventually lyse the infected host cell and disseminate infection. Stable transfection and drug selection are performed in the stage (the lightning bolt symbolizes electroporation). Once stable genomic changes are confirmed in epimastigotes, these parasites are used to establish the mammalian infection cycle starting with metacyclic trypomastigotes as outlined above. Download FIG S1, TIF file, 2.3 MB. Copyright © 2023 Won et al. 2023 Won et al. https://creativecommons.org/licenses/by/4.0/ This content is distributed under the terms of the Creative Commons Attribution 4.0 International license.

In both insect and mammalian hosts, T. cruzi can establish intimate contact with host structures using its single flagellum (3–5). In triatomine vectors, epimastigotes attach to the hindgut by forming a hemidesmosome-like structure between the distal part of the flagellum and host rectal epithelium (5). This attachment prevents the epimastigotes from being flushed from the insect and is important for promoting differentiation to the infectious metacyclic stage (5). In mammalian host cells, cytosolically localized T. cruzi amastigotes establish intermittent contact with host mitochondria using their short motile flagellum (3, 6). Unlike the motile trypomastigote and epimastigote stages of T. cruzi, which have elongated flagella (up to 15 μm in length [7]), replicative intracellular amastigotes have a truncated flagellum (~2.7 μm) that extends just beyond the opening of the flagellar pocket (6). Also, T. cruzi amastigotes retain a 9 + 2 axonemal structure found in motile trypanosomatid life stages (8) but lack a paraflagellar rod, a unique lattice-like structure which runs parallel to the axoneme in these organisms (9) and which is associated with several functions, including flagellar motility and signal transduction (10). It has long been assumed that the minimal amastigote flagellum has no function other than to provide a structural platform for flagellar outgrowth during differentiation to motile life stages (11). However, recent observations that the flagellum of intracellular T. cruzi amastigotes exhibits low-frequency aperiodic “beating” inside mammalian host cells (6) and makes physical contact with the host mitochondria (3, 6) indicate that the amastigote flagellum has a functional role within the host cell. The interaction between the T. cruzi amastigote flagellum and host mitochondria is comparable to the intimate contact observed between the flagellum of intracellular Leishmania mexicana amastigotes and the host parasitophorous vacuole membrane (12). In the case of *Leishmania*, it has been postulated that the amastigote flagellum has a sensory role and/or functions in the delivery of parasite material to the infected host cell (12, 13). It is therefore reasonable to predict that the T. cruzi amastigote flagellum may have a similar role(s) in its interactions with the intracellular environment of the host cell.

In addition to critical roles in motility, eukaryotic flagella and cilia, as well as nonmotile cilia, have emerged as important sensory organelles that are equipped with signal transduction systems and second messengers such as cyclic AMP (cAMP) (14) and calcium (15, 16) that coordinate cellular responses to external stimuli. Functions beyond cell locomotion have also been ascribed to the flagellum of motile life stages of trypanosomatid protozoans (11, 13, 17–19). The best-understood example of sensory integration in these organisms is the role of flagellar receptor-type adenylate cyclases and cAMP-dependent signaling in pH taxis and social motility in the insect stage of Trypanosoma brucei (20–23). In *Leishmania*, flagellar aquaporin has been implicated in osmotaxis (24) in the insect stage, and the flagellar membrane may be a critical site for glucose (25, 26) and arginine (27) sensing in these parasites. Indeed, in both T. brucei and *Leishmania*, nearly comprehensive flagellar proteomes have been generated using shotgun proteomics of isolated flagella (28–30) or of detergent- and high-salt-extracted fractions of the flagellum yielding axonemal and paraflagellar rod proteins (31). Further, in T. brucei, specific domains of the flagellum have been partially mapped using proximity-dependent biotinylation, including flagellar attachment zone proteins (32) and the flagellar tip (33), a specialized signaling domain.

In comparison, we have little knowledge of the molecular composition of the T. cruzi flagellum. Beyond a core axonemal proteome that is predicted based on conservation across trypanosomatid species and life stages (34), few flagellar proteins with the potential to serve as a functional interface with the host environment have been identified in any T. cruzi life stage (9, 35, 36). The best-characterized nonaxonemal flagellar protein in T. cruzi is the flagellar calcium-binding protein (FCaBP), a dual-acylated, 24-kDa Ca^2+^-sensing protein that is tethered to the inner leaflet of the flagellar membrane (37). FCaBP is expressed in all T. cruzi life stages and is conserved across other trypanosomatid species. It is predicted to function as a calcium sensor, but its precise role in the biology of these organisms is unknown (36, 38, 39). Additionally, we recently showed that the T. cruzi small myristoylated protein 1-1 (TcSMP1-1) localizes nearly exclusively to the flagellum of T. cruzi amastigotes (6). Apart from these examples, the proteomic landscape of the T. cruzi flagellum remains largely uncharacterized.

In this study, we pursued a targeted, proximity-dependent biotinylation (BioID) approach to identify flagellar membrane and membrane-proximal flagellar proteins in the replicative stages of T. cruzi. We report the identification of 218 and 99 candidate flagellar proteins in T. cruzi epimastigotes and intracellular amastigote stages, respectively, many of which are conserved in other trypanosomatid species with evidence of flagellar localization. Approximately 20% of the candidate flagellar proteins were found to be restricted to the T. cruzi lineage, including a hypothetical protein that we confirmed localizes to the flagellar tip in T. cruzi epimastigotes and intracellular amastigotes. The novel BioID data set identified here provides a critical foundation for investigation of the T. cruzi flagellum and its role in mediating interactions with diverse host environments.

## RESULTS

### Flagellar and cytosolically targeted TurboID retains activity in T. cruzi.

**FIG 1:**
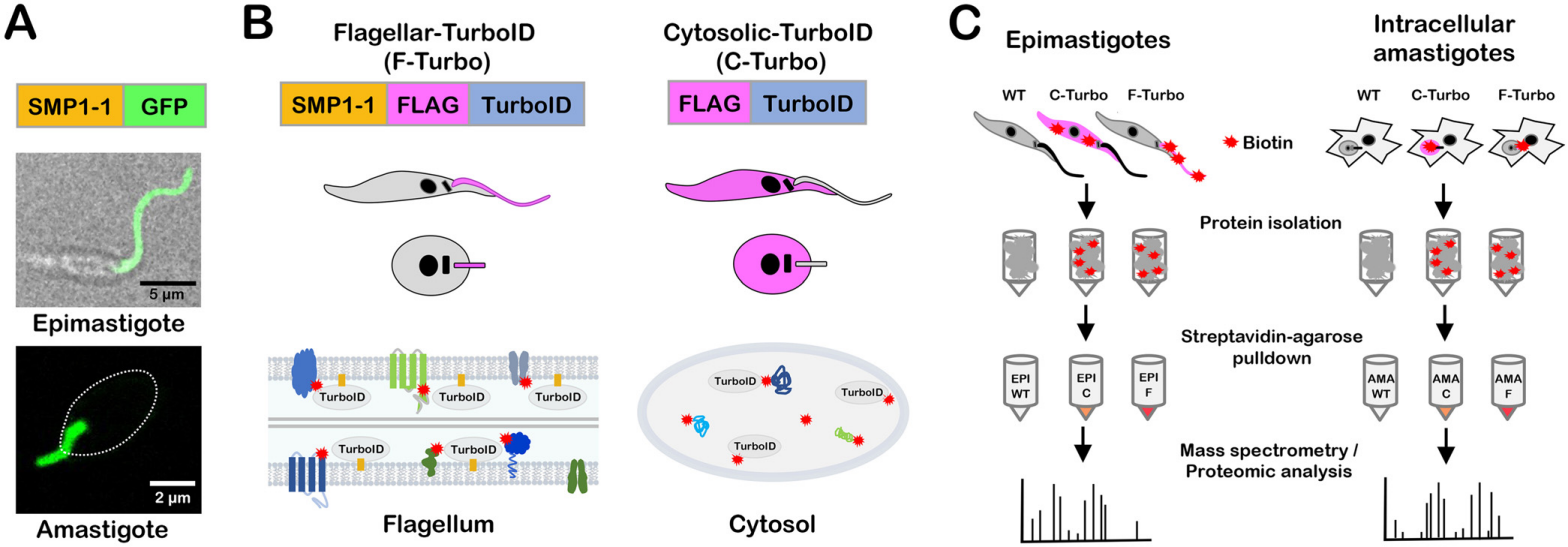
T. cruzi life cycle and schematic of TurboID-expressing lines generated for proximity-dependent biotinylation experiments. (A) Live confocal images of SMP1-1–GFP localized to the flagellum of T. cruzi epimastigotes and an intracellular amastigote; the white oval denotes the position of the amastigote body. (B) Strategy for generating stable T. cruzi lines expressing TurboID in the flagellum using SMP1-1 as the endogenous bait protein or in the cytoplasm of epimastigotes and amastigotes, where addition of exogenous biotin mediates the biotinylation (red star) of proteins in close proximity to TurboID in both settings. The FLAG epitope is included to facilitate TurboID localization in transfectants. (C) Flow chart outlining the experimental protocol used for identification of biotinylated proteins in epimastigotes (left) and intracellular amastigotes (right). For both life stages, wild-type (WT), cytoplasmic-TurboID (labeled “C”), and flagellar-TurboID (labeled “F”) parasites (left to right) were exposed to biotin, and the biotinylated protein fractions in protein lysates were captured on streptavidin-agarose beads and subjected to mass spectrometry for identification and subsequent proteomic analysis.

To facilitate the identification of flagellar proteins in T. cruzi using a proximity-dependent biotinylation approach, we generated transgenic parasites that express the biotin ligase TurboID (40) in the parasite flagellum, as an in-frame fusion with C-terminally FLAG-tagged TcSMP1-1 (SMP1-1-FLAG-TurboID [F-Turbo]) (Fig. 1A and B). TcSMP1-1 was chosen as the endogenous bait protein for flagellar localization of TurboID given its nearly exclusive localization in the flagellum in both replicative stages of T. cruzi, epimastigotes and amastigotes (6) (Fig. 1A), and because TcSMP1-1 contains the N-myristoylation sequence motif (MGXXXS/T) required for localization and tethering to the inner flagellar membrane, as demonstrated in *Leishmania* (41). The strategy of targeting TurboID to the flagellum using TcSMP1-1 is expected to increase the likelihood of identifying flagellar membrane and associated proteins while minimizing capture of axonemal proteins. To control for nonflagellar TurboID expression in the F-Turbo parasites, we generated an independent transgenic line that expresses FLAG-TurboID in the cytoplasm (C-Turbo) (Fig. 1B). The parallel processing of F-Turbo and C-Turbo parasites, along with parental (wild-type [WT]) parasites that lack TurboID expression (Fig. 1C), allows background subtraction and identification of proteins enriched in the flagellum in F-Turbo versus C-Turbo parasites within the same life cycle stage.

**FIG 2:**
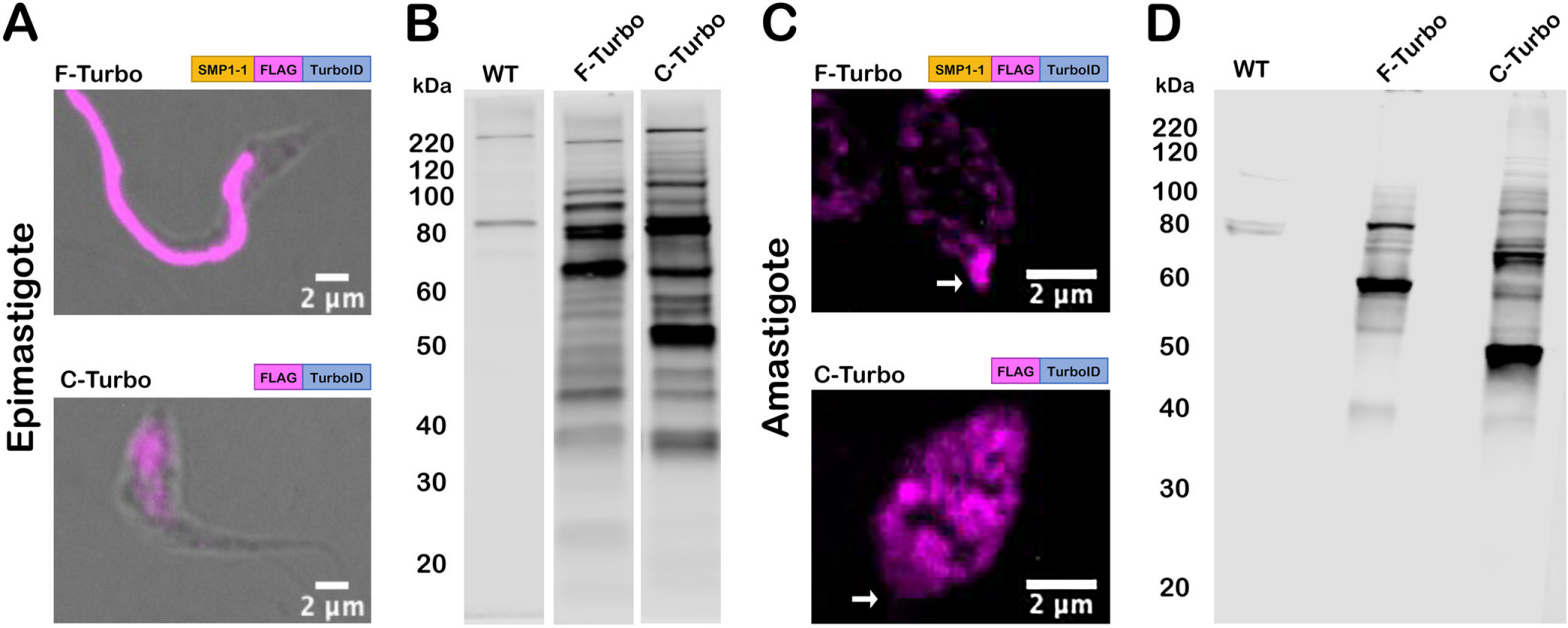
TurboID localization and activity in T. cruzi. (A and C) Fluorescence microscopy images of fixed T. cruzi epimastigotes (A) or amastigotes (C) expressing SMP1-1-FLAG-TurboID (F-Turbo) (top) or FLAG-TurboID (C-Turbo) (bottom) stained for FLAG epitope (anti-FLAG) (pink). In panel C, white arrows indicate the position of the amastigote flagellum. (B and D) Biotinylated proteins in lysates of WT, F-Turbo, and C-Turbo T. cruzi epimastigote (B) and amastigotes (D) detected with streptavidin-DyLight 800.

TurboID expression in transgenic T. cruzi parasites was confirmed by indirect immunofluorescence microscopy of fixed parasites stained with an antibody to the FLAG tag epitope, located immediately upstream of TurboID (Fig. 2). The flagella of F-Turbo parasites were brightly stained (Fig. 2A and C, F-Turbo panels), indicating that trafficking of TurboID to the flagellum occurred in both T. cruzi epimastigote (Fig. 2A, F-Turbo panel) and amastigote (Fig. 2C, F-Turbo panel) life stages. While most of the FLAG signal was localized to the flagellum in epimastigotes (Fig. 2A, F-Turbo panel), signal was detected in the body of intracellular amastigotes in addition to the brightly stained flagellum (Fig. 2C, F-Turbo panel), which may be due to overexpression of the SMP1-1-FLAG–TurboID fusion protein. C-Turbo parasites, generated as a proteomic control for nonflagellar TurboID-dependent biotinylation (Fig. 1C), were confirmed to express cytosolic FLAG in both parasite life stages (Fig. 2A and C, C-Turbo panels). To determine if TurboID is active in T. cruzi, total protein lysates were prepared from WT and Turbo-expressing parasites, following brief exposure to exogenous biotin, were probed with streptavidin-DyLight 800 to detect biotinylated proteins (Fig. 2B and D). As expected, multiple biotinylated proteins were revealed in lysates derived from F-Turbo and C-Turbo epimastigotes (Fig. 2B) and amastigotes (Fig. 2D), whereas few biotinylated proteins were detected in the parental (WT) controls. Differences in the biotinylated protein profiles observed when F-Turbo parasites were compared to C-Turbo parasites within a single life stage (Fig. 2B and D) likely reflect the differential localization patterns for TurboID in these parasite lines. Combined, these results confirm the expression of active TurboID in the flagellum (F-Turbo) or cytosol (C-Turbo) in both replicative stages of T. cruzi.

### Proteomic identification of candidate flagellar proteins in T. cruzi.

**FIG 3:**
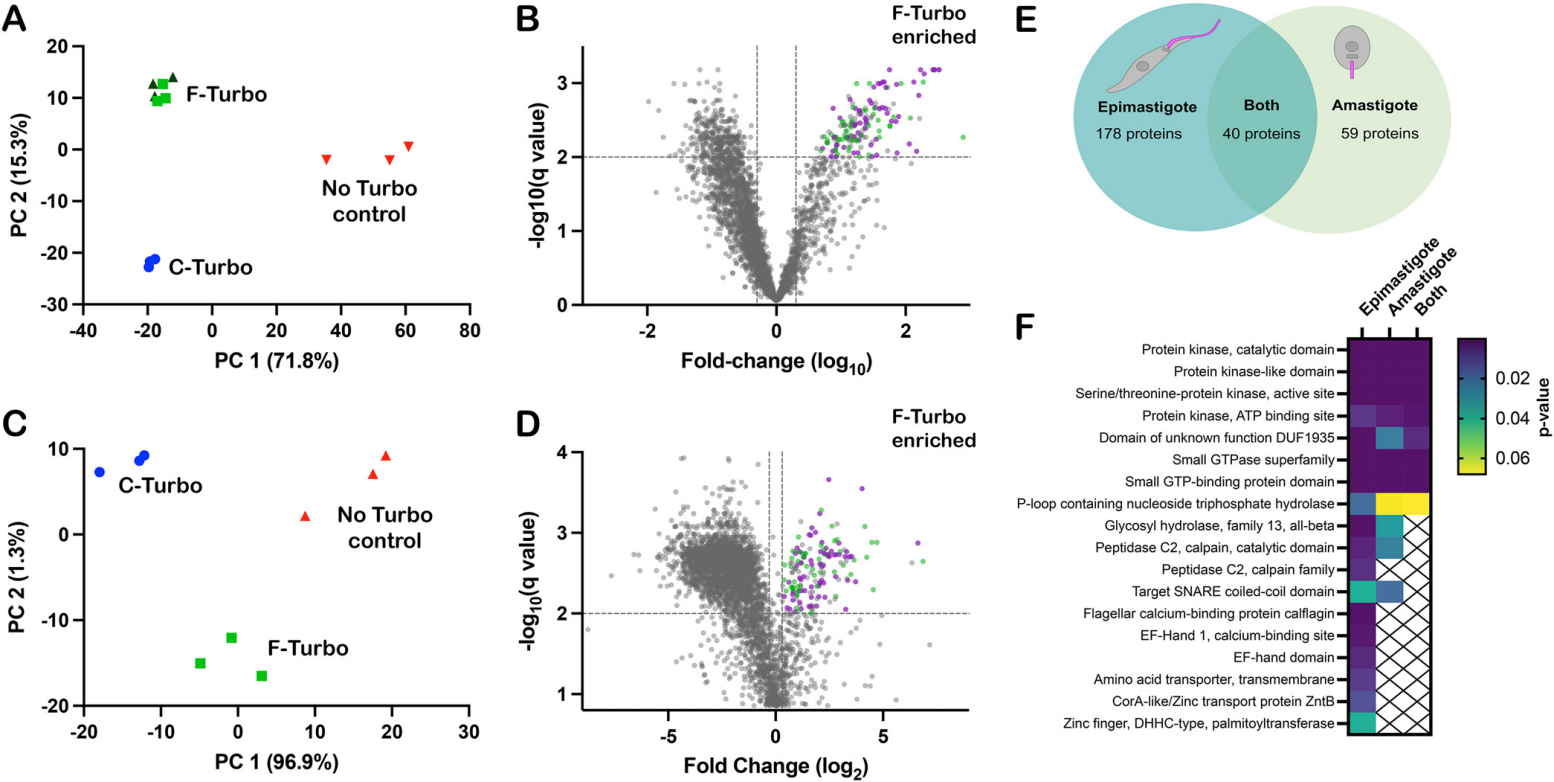
Proximity proteome analysis identifies enriched flagellar proteins in T. cruzi. Principal-component analysis (PCA) of biotinylome data plotted for WT (no TurboID control), flagellar-TurboID (F-Turbo) and cytosolic-TurboID (C-Turbo) for T. cruzi epimastigotes (A) and intracellular amastigotes (C). The two independent F-Turbo groups in the epimastigote PCA plot are represented in green (triangles and squares). Volcano plots (B and D) with fold change (F-Turbo versus C-Turbo; *x* axis) and adjusted *P* value (*q* value; *y* axis) for T. cruzi epimastigote (B) and amastigote (D) proteomic data. Horizontal lines represent a *q* value of 0.01, and the two vertical lines indicate the cutoffs for fold change (2-fold). The top right quadrants in each plot (B and D) contain proteins that are significantly enriched in F-Turbo proteomes (*q* ≤ 0.01, ≥2-fold change). Known trypanosomatid flagellar proteins (purple circles) and hypothetical proteins (green circles) are shown for the F-Turbo enriched proteins. (E) Venn diagram depicting the number of proteins identified as enriched in the F-Turbo samples of T. cruzi epimastigote and amastigote stages. (F) Interpro domains assigned by DAVID that are significantly enriched in F-Turbo samples (i.e., proteins found in the upper right quadrant of each volcano plot) in epimastigotes and amastigotes and those common to both life stages. *P* values are from a modified Fisher exact test for protein enrichment analysis.

Biotinylated proteins in lysates generated from WT, F-Turbo, and C-Turbo T. cruzi epimastigotes or intracellular amastigotes were captured on immobilized streptavidin beads and identified using high-performance liquid chromatography combined with mass spectrometry (HPLC-MS) (Fig. 1C). Three independent biological replicates were analyzed for each parasite line, with the exception of F-Turbo epimastigotes, for which triplicate samples from two independent transfections were included. Peptide identification and relative intensity data obtained for replicate samples from each parasite line are presented in Table S1. Principal-component analysis (PCA) identified overall trends in the proteomic data obtained for T. cruzi epimastigotes (Fig. 3A) and amastigotes (Fig. 3C), revealing that biological replicates from individual parasite lines (WT, F-Turbo, and C-Turbo) formed discrete clusters that were well separated from each other. As the replicates from independent F-Turbo epimastigote lineages were indistinguishable, these samples were pooled for subsequent analyses. Prior to data filtering and analysis, protein intensity scores were averaged across biological replicates within individual experimental groups (Table S1).

10.1128/msphere.00088-23.4 TABLE S1 Raw proteomics data for the epimastigotes and intracellular amastigotes in the TurboID experiment. Each sheet contains the protein identity and intensity score for all samples in either epimastigotes or amastigotes. Download Table S1, XLSX file, 1.6 MB. Copyright © 2023 Won et al. 2023 Won et al. https://creativecommons.org/licenses/by/4.0/ This content is distributed under the terms of the Creative Commons Attribution 4.0 International license.

Streptavidin-bound proteins identified by mass spectrometry in WT parasites (which lack TurboID) represent the background signal of endogenously biotinylated proteins and proteins that bound nonspecifically to immobilized streptavidin. Thus, proteins that were <100-fold enriched in F-Turbo or C-Turbo epimastigote samples relative to the WT samples were removed before subsequent analysis. Also, any proteins identified in fewer than 4/6 F-Turbo samples were removed (Table S2). Volcano plots revealed the protein subsets significantly enriched in F-Turbo and C-Turbo samples in epimastigotes (Fig. 3B) and amastigotes (Fig. 3D). Proteins found to be significantly enriched in F-Turbo samples over C-Turbo samples (fold change > 2; *q* value ≤ 0.01) as well as proteins identified uniquely in F-Turbo samples (i.e., not present in C-Turbo samples from the same parasite life stage) are listed in Table S3. From this analysis, 218 proteins were identified as significantly enriched in F-Turbo samples from T. cruzi epimastigotes and 99 proteins in amastigotes (Table S3).

10.1128/msphere.00088-23.5 TABLE S2 Filtered proteomics data for the epimastigote TurboID experiment. This sheet contains the protein identity and intensity score for all samples in epimastigotes that were used for statistical analysis. Download Table S2, XLSX file, 0.6 MB. Copyright © 2023 Won et al. 2023 Won et al. https://creativecommons.org/licenses/by/4.0/ This content is distributed under the terms of the Creative Commons Attribution 4.0 International license.

10.1128/msphere.00088-23.6 TABLE S3 Proteins enriched in the flagellar TurboID expressing samples for epimastigotes and intracellular amastigotes. Each sheet contains information about the proteins enriched in either epimastigotes, amastigotes, or both. Proteins are presented in ascending order based on *q* value; the table for both epimastigote and amastigote F-Turbo enriched proteins presents data in ascending order based on epimastigote *q* value. Download Table S3, XLSX file, 0.05 MB. Copyright © 2023 Won et al. 2023 Won et al. https://creativecommons.org/licenses/by/4.0/ This content is distributed under the terms of the Creative Commons Attribution 4.0 International license.

### The T. cruzi SMP1-1 proximity proteome includes known trypanosomatid flagellar proteins.

The searchable TrypTag database (42), which contains localization data for 7,487 T. brucei proteins, was used as a resource to identify orthologues in the data set of T. cruzi enriched flagellar proteins (Table S3) that have demonstrated flagellar localization in T. brucei. Of the 218 enriched flagellar proteins in T. cruzi epimastigotes, 145 have orthologs that are represented in the TrypTag database, and of these, 75 proteins exhibit at least partial flagellar localization in T. brucei bloodstream forms (42). Similar results emerged from the T. cruzi amastigote data, where orthologs of 75 of the 99 proteins found to be enriched in amastigote F-Turbo samples had orthologs in T. brucei and were endogenously tagged, and 44 of these showed at least partial flagellar localization. A comparison of the enriched flagellar proteins identified in both T. cruzi epimastigotes and amastigotes revealed 40 proteins common to both life stages (Fig. 3E), of which 29 have orthologs that are represented in the TrypTag database (Table S3), and 20 proteins exhibited some flagellar localization in T. brucei. Examples of confirmed flagellar proteins in other trypanosomatids that are significantly enriched in the T. cruzi flagellar proximity proteome include flagellar membrane 8 (43), flabarin (44), flagellar attachment zone 14 (32), casein kinase I (42), CARP3 (20), and cysteine peptidase, clan CA, family C2 (calpain 1.3) (42). Although a significant proportion of the flagellar candidates identified in T. cruzi epimastigotes and amastigotes fall into the “hypothetical” category (i.e., no annotation), the data sets were found to be enriched in kinase domains, calpain domains, and small GTP binding protein/GTPase domains (Fig. 3F).

### Selected flagellar candidates localize to the T. cruzi flagellum in epimastigotes and amastigotes.

**FIG 4:**
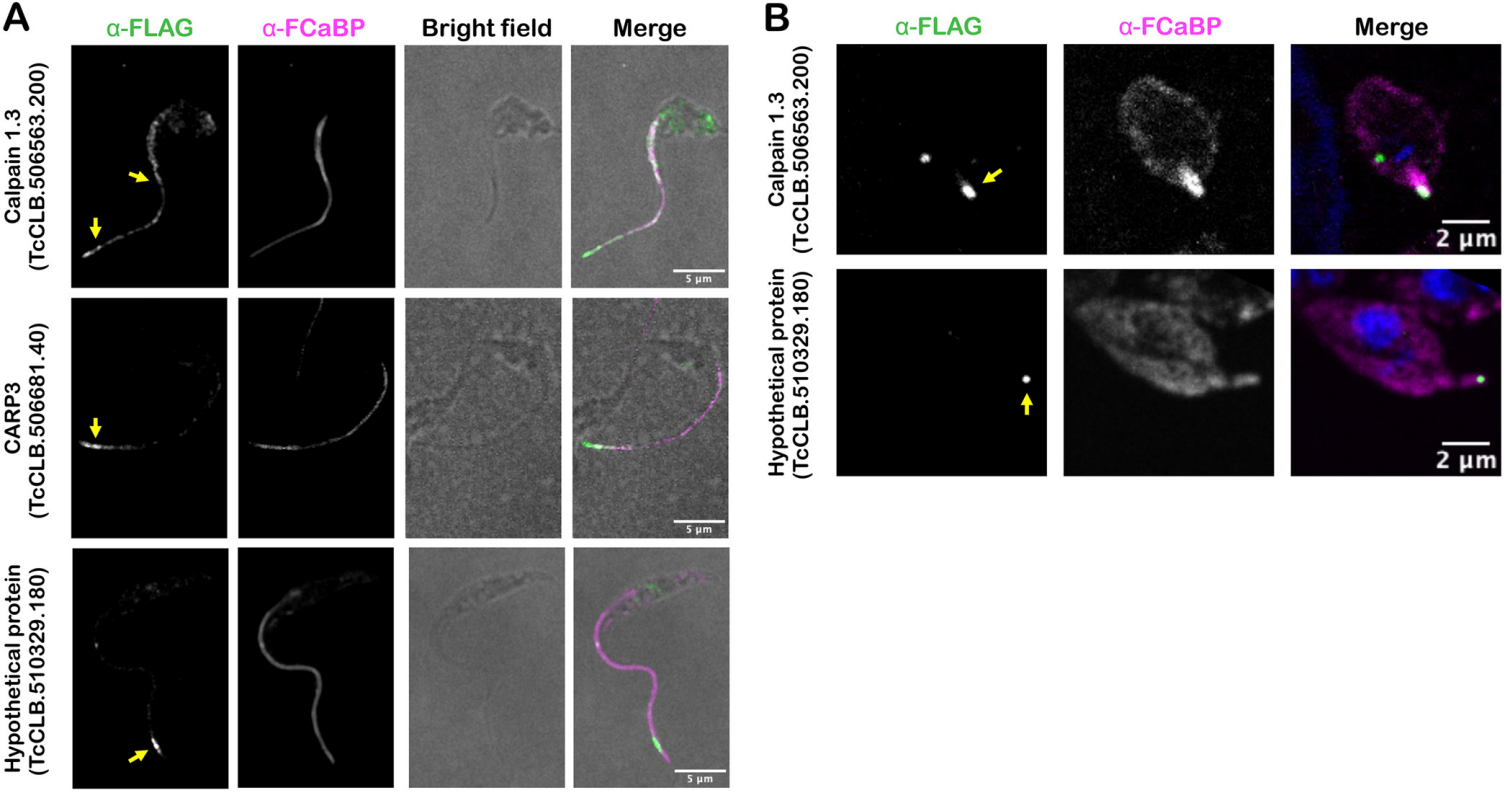
Flagellar localization of candidate flagellar proteins in T. cruzi. Endogenous tagging reveals flagellar localization of candidate flagellar proteins: calpain 1.3-smFLAG (TcCLB.506563.200), CARP3-smFLAG (TcCLB.506681.40), or hypothetical protein-FLAG (TcCLB.510329.180) in T. cruzi epimastigotes (A) or intracellular amastigotes (B). In all cases, the FLAG tag was detected in fixed parasites using an anti-FLAG antibody and secondary antibody (green) and the flagellum was detected using anti-FCaBP and secondary antibody (magenta). The FLAG signal in the flagellum is indicated (yellow arrow).

To localize candidate flagellar proteins in T. cruzi, we prioritized those that were significantly enriched in both the epimastigote and amastigote F-Turbo data sets or were found only in the amastigote F-Turbo data set and that included one or more of the following characteristics: (i) sequence motifs predicting membrane localization, (ii) a predicted role in signaling based on annotation, and (iii) unique presence in the T. cruzi lineage (i.e., no obvious orthologues in other trypanosomatid species). Based on these criteria, six proteins were selected for endogenous FLAG tagging and subsequent subcellular localization (Table 1) using primers and homology-directed repair templates shown in Table S4. Four of the 6 proteins were successfully tagged, and three of these exhibited flagellar localization in T. cruzi epimastigotes: calpain 1.3 (TcCLB.506563.200), CARP3 (TcCLB.506681.40), and a hypothetical protein (TcCLB.510329.180) (Fig. 4A; Fig. S2). Another hypothetical protein (TcCLB.509965.20) was not verified as flagellar as the FLAG epitope signal localized to the parasite body (data not shown). Calpain 1.3-mRuby2-smFP FLAG exhibited a punctate pattern of labeling along the entire length of the T. cruzi epimastigote flagellum (Fig. 4A), whereas expression appeared to be restricted to the flagellar tip in the intracellular amastigote stage (Fig. 4B). Both CARP3 and the hypothetical protein (TcCLB.510329.180) localized to the distal region of the epimastigote flagellum (Fig. 4A), and the hypothetical protein (TcCLB.510329.180) also localized to the flagellar tip in amastigotes (Fig. 4B). We were unable to determine CARP3 localization in amastigotes, due to undetectable signal for CARP3-mRuby2-smFP FLAG expression in this life stage, despite a clear signal and flagellar localization in epimastigotes (Fig. 4A). Nonetheless, the successful identification of a subset of flagellum-localized proteins in intracellular T. cruzi amastigotes and epimastigotes provides initial validation of the proteomic data sets generated from proximity-dependent labeling.

**TABLE 1:** Candidate flagellar proteins in T. cruzi selected for endogenous epitope tagging and localization

Name	TriTrypDB ID	Successfully tagged	Reason for selection	F-Turbo/C-Turbo	
				Epimastigote	Amastigote
Calpain 1.3	TcCLB.506563.200	Yes	Potential signaling role	189.84	9.88
CARP3	TcCLB.506681.40	Yes	Potential signaling role	59.87	10.96
Hypothetical protein	TcCLB.510329.180	Yes	No known ortholog	69.36	4.37
Hypothetical protein	TcCLB.509965.20	Yes	No known T. brucei ortholog	2,953.04	22.34
ATPase	TcCLB.506925.410	No	Potential transmembrane protein		6.05
Hypothetical protein	TcCLB.509011.50	No	Potential signaling role	49.20	2.2

10.1128/msphere.00088-23.2 FIG S2 PCR confirmation of endogenous tags for candidate proteins. (A) Schematic showing the region of amplification and DNA gel with corresponding bands for calpain 1.3-smFLAG (TcCLB.506563.200) (A), CARP3-smFLAG (TcCLB.506681.40) (B), and hypothetical protein-FLAG (TcCLB.510329.180) (C). The DNA ladder is shown with sizes (in base pairs) on the left. Download FIG S2, TIF file, 1.0 MB. Copyright © 2023 Won et al. 2023 Won et al. https://creativecommons.org/licenses/by/4.0/ This content is distributed under the terms of the Creative Commons Attribution 4.0 International license.

## DISCUSSION

In the present work, we demonstrate the successful use of a proximity labeling tool in the protozoan parasite Trypanosoma cruzi. With the goal of identifying flagellar membrane and/or associated proteins in T. cruzi as candidates for mediating physical or functional interactions with insect or vertebrate hosts, the biotin ligase TurboID was targeted to the parasite flagellum as a fusion protein with the inner flagellar membrane protein SMP1-1. Overexpression of the fusion protein was well tolerated in the parasite, with no interference in the ability to transition between axenic epimastigotes, the T. cruzi life stage in which DNA transfection and selection are performed, and the intracellular stages in mammalian cells. This offered the opportunity to perform a comparative analysis of the two main replicative stages of T. cruzi, one that is motile with an elongated flagellum (epimastigote) and one that is nonmotile with a short flagellum (intracellular amastigote). Furthermore, inclusion of cytosolic-TurboID-expressing parasites in the analysis aided in the differential identification of enriched flagellar biotinylated proteins derived from SMP1-1-FLAG-TurboID parasites. This approach yielded 218 flagellar candidates in epimastigotes and 99 proteins in amastigotes, where 40 proteins were common to both T. cruzi life stages. Flagellar localization was confirmed for a subset of proteins in this data set, based on endogenous epitope tagging, and many more were predicted based on demonstrated localization in the related trypanosomatids T. brucei and *Leishmania* spp.

The functional capabilities of the T. cruzi flagellum are broadly uncharacterized, beyond its role in propelling motile life stages and anchoring epimastigotes to the rectal mucosa in the insect vector (4, 45, 46). However, the recent recognition that the flagellum of cytosolically localized intracellular amastigotes is capable of beating and establishes physical contact with host mitochondria (3, 6) points to a potential role for the amastigote flagellum in host environmental sensing. Although little is known regarding the sensory capabilities of trypanosomatids in general, significant progress has been made toward a molecular understanding of pH taxis and social motility in the insect stages of T. brucei (21, 23). The sensory system involves regulation of cyclic AMP levels, modulated by flagellar receptor-type adenylate cyclases and cyclic AMP phosphodiesterases (22) and the involvement of a cyclic AMP-responsive protein (CARP3), which is thought to act in a complex with adenylate cyclases (20). Our discovery that CARP3 is expressed in the replicative stages of T. cruzi, including intracellular amastigotes (despite the inability to localize the tagged protein in this life stage), is quite exciting given the established role of CARP3 in T. brucei (20). In T. brucei, CARP3 is known to colocalize with calpain 1.3 at the distal region of the flagellum, where the two proteins may physically interact (20). We show that the calpain 1.3 ortholog is also expressed in the flagellum of T. cruzi, where it localizes exclusively to the flagellar tip in intracellular amastigotes, a recognized signaling domain in trypanosomatids (p2) (33, 47). Calpain 1.3 belongs to a subfamily of cysteine peptidases that are predicted to be catalytically inactive, as they lack one or more of the active-site amino acid residues (48). Proteins with these features are thought to play a role in calcium homeostasis/signaling, including the calcium-based regulation of adenylate cyclase complexes (20, 49). Despite the lack of social motility in T. cruzi, the expression of flagellar CARP3 and calpain 1.3 in this species is a strong indicator that the T. cruzi flagellum is equipped to sense and integrate signals from the environment. Dissection of the functional roles of CARP3 and calpain 1.3 in T. cruzi is expected to be instrumental in establishing the existence of flagellum-based environmental sensing in this parasite. In addition, a functional investigation of the hypothetical protein (TcCLB.509965.20) that also localizes to the distal end of the T. cruzi flagellum but lacks an obvious ortholog in T. brucei or *Leishmania* has the potential to reveal novel biological or mechanistic insights into the role of the T. cruzi flagellum in different life stages.

The proteomic data sets generated here offer new opportunities to pursue the localization and functional analyses of many uncharacterized proteins, some of which are unique to the T. cruzi lineage. While some of the annotated proteins are not predicted to localize to the flagellum (based on annotation and TrypTag localization), including proteins involved in protein trafficking which may have encountered SMP1-1-FLAG-TurboID en route to the flagellum, there are a number of proteins with transmembrane domains or N-myristoylation consensus sequences that predict membrane association. SMP-1 contains the N-myristoylation sequence motif (MGXXXS/T) known to direct flagellar localization and tethering to the inner flagellar membrane in *Leishmania*, where it associates tightly with detergent-resistant membranes (lipid rafts) (41) and forms homodimers (41). As a result, SMP1-1 may preferentially interact with other membrane proteins associated with lipid rafts in the flagellar membrane. It is notable that a number of proteins that were among the strongest hits in the flagellar candidate pool, such as calpain 1.3 and CARP3, also have MGXXXS/T motifs. While this motif is insufficient to direct a protein to the flagellum (41), proteins with lipid anchoring motifs or transmembrane domains could be prioritized for future studies of the T. cruzi flagellum. Notably, we did not identify adenylate cyclases in our data, even though CARP3 and calpain 1.3 were identified in a proximity-labeling study in T. brucei designed to identify flagellar tip proteins that interact with adenylate cyclase 1 (33). While the proximity-dependent labeling approach used in this study enabled the discovery of a subset of flagellar proteins in T. cruzi amastigotes (where physical isolation of the short amastigote flagellum may not be feasible), it is understood that the resulting proteomes derived for the two parasite life stages are not comprehensive and that many T. cruzi flagellar membrane proteins remain to be identified. With the discovery of additional flagellar proteins in T. cruzi, opportunities to use one or more of these proteins as alternative bait proteins for proximity labeling will arise, with a view to expanding the flagellar proteome in this understudied parasite.

Overall, we have presented the first use of proximity-dependent biotinylation in T. cruzi for the identification of more than 200 candidate flagellar proteins across two parasite life stages, thereby creating an important resource for the research community. As more information becomes available for the T. cruzi amastigote flagellum, it is expected to provide some context for the biological role of the flagellum in infection, and these interactions may be the key for specific targeting of parasite function and viability within the mammalian host. Future investigation focused on identifying the function of potential sensory flagellar candidates in T. cruzi epimastigote and amastigote flagella may aid in the discovery of these currently unknown host-parasite interaction mechanisms.

## MATERIALS AND METHODS

### Reagents.

Compounds were purchased and diluted to stock concentrations: biotin, 100 mM in dimethyl sulfoxide (DMSO) (Sigma-Aldrich, St. Louis, MO, USA); phenylmethylsulfonyl fluoride (PMSF), 10 mM in isopropanol (Sigma-Aldrich, St. Louis, MO, USA); and tosyl-l-lysyl-chloromethane hydrochloride (TLCK), 5 mM in DMSO (Abcam, Cambridge, United Kingdom).

### Mammalian cell culture.

Normal human neonatal dermal fibroblasts (NHDF; Lonza, Basel, Switzerland) and monkey kidney epithelial cells (LLC-MK2; American Type Culture Collection) were maintained in Dulbecco’s modified Eagle medium (DMEM; HyClone, Logan, UT, USA) supplemented with 10% heat-inactivated fetal bovine serum (FBS) (Gibco, Waltham, MA, USA), 25 mM glucose, 2 mM l-glutamine, and 100 U/mL penicillin-streptomycin (DMEM-10) at 37°C and 5% CO_2_.

### Growth and maintenance of T. cruzi.

Trypanosoma cruzi Tulahuen LacZ clone C4 was obtained from the American Type Culture Collection (ATCC) (PRA-330; ATCC, Manassas, VA, USA). The epimastigote stage was propagated at 28°C in liver infusion tryptose (LIT) medium (4 g/L NaCl, 0.4 g/L KCl, 8 g/L Na_2_HPO_4_, 2 g/L dextrose, 3 g/L liver infusion broth, 5 g/L tryptose, with 25 mg/L hemin and 10% heat-inactivated FBS). The mammalian cell infection cycle was initiated with metacyclic trypomastigotes arising within stationary-phase epimastigote cultures that were shifted from LIT medium to DMEM containing 2% FBS (DMEM-2) for 5 days at 28°C. Metacyclic stage-enriched cultures were washed in DMEM-2 and incubated with confluent LLC-MK2 monolayers at 37°C and 5% CO_2_ to allow invasion. Mammalian-stage trypomastigotes that emerged from infected LLC-MK2 cells (within 5 to 10 days) were harvested from culture supernatants and used to infect fresh LLC-MK2 monolayers. This cycle was continued on a weekly basis to maintain the mammalian-cell-infective stages of T. cruzi in culture. For experimental infections, trypomastigotes collected from LLC-MK2 maintenance cultures were pelleted at 2,060 × *g* for 10 min, and pellets were incubated at 37°C and 5% CO_2_ for 2 to 4 h to allow motile trypomastigotes to swim up into the supernatant. Purified trypomastigotes in the supernatant were collected, washed once in DMEM-2, and utilized to infect subconfluent monolayers of NHDF as indicated.

### Generation of TurboID-expressing T. cruzi lines.

T. cruzi strains expressing TcSMP1-1 fused to green fluorescent protein (TcSMP1-1–GFP) were previously generated (6). A plasmid containing the TurboID sequence was a kind gift from Jeffrey Dvorin (Harvard Medical School). Each TurboID construct was used to replace the GFP-P2A-puro cassette in a modified pTREX plasmid (50, 51) containing either SMP1-1–GFP or GFP alone. The inserts in the pTREX backbone to generate the F-Turbo plasmids were SMP1-1-TurboID-P2A-puro (F-Turbo-P) and SMP-1-1-TurboID-T2A-puro (F-Turbo-T). The inserts and backbone were assembled using the NEBuilder HiFi DNA assembly kit (New England Biolabs, Ipswich, MA, USA), resulting in the final plasmids. For the cytosolic control, TurboID-P2A-puro was amplified using PCR and then inserted into the pTREX-GFP backbone, replacing GFP between the SpeI and XmaI cut sites using restriction enzyme cloning. To generate TurboID-expressing parasites, T. cruzi epimastigotes were transfected with 15 μg of the respective DNA. Prior to transfection, log-phase T. cruzi epimastigotes were pelleted at 2,060 × *g* for 10 min, resuspended in 100 μL of TbBSF buffer (52) (4 × 10^7^ parasites), placed in a sterile 2-mm-gap cuvette with the appropriate DNA, and transfected using an Amaxa Nucleofector II device (Lonza, Basel, Switzerland; U-33 program). Parasites were immediately transferred to LIT medium for 24 h before addition of 10 μg/mL puromycin (InVivogen, San Diego, CA, USA) or 50 μg/mL blasticidin (InVivogen, San Diego, CA, USA) for continuous selection. Epimastigote populations expressing SMP1-1-FLAG-TurboID (F-Turbo) were cloned via limiting dilution (51), and clones were screened by indirect immunofluorescence (see below) to detect and localize the FLAG epitope. Clonal populations exhibiting >75% FLAG-positive flagella were expanded and used for further analysis. Given that ~75% of parasites in bulk populations of FLAG-TurboID-expressing parasites (C-Turbo) were FLAG positive, the bulk population was used for further analysis.

### Endogenous epitope tagging of candidate flagellar proteins.

CRISPR/Cas9-facilitated epitope tagging of genomic loci in T. cruzi was performed as described previously (53). Briefly, each gene of interest was PCR amplified from genomic DNA (T. cruzi Tulahuen strain), and PCR products were sequenced. Two guide RNA binding sites near the 3′ region of each gene of interest were identified using EuPaGDT (54). Editing of the previously modified pTREX-n-Cas9 plasmid (51) (Addgene plasmid 68708), performed to exchange the previous gRNA sequence, was achieved using a Q5 mutagenesis kit (New England Biolabs, Ipswich, MA, USA). gRNA sequences were inserted into pTREX-n-Cas9 using primers specific to the gene of interest (Table S4), such that the previous gRNA sequence was replaced. The template for generating homology-directed repair DNA for gene tagging was constructed by inserting a P2A viral skip peptide in frame with a downstream blasticidin-S deaminase (BSD) or puromycin *N*-acetyl-transferase (puro) and TOPO cloned into a pCR4 backbone (Thermo Fisher, Waltham, MA, USA) (51). The homology template was amplified from this template using ultramer pairs (Table S4) that provided 100 bp of homology for the gene of interest, the FLAG tag, and 20 bp of homology to template. Parasite were transfected as described above with 25 μg of each gRNA-specific pTREX-n-Cas9 plasmid to the gene of interest and 50 μg of homology repair template. Correct integration of the endogenous tag and drug cassette was established via PCR (Fig. S2).

10.1128/msphere.00088-23.7 TABLE S4 Primers for all PCR and endogenous tagging. Primer pairs are listed for all experiments described. Download Table S4, XLSX file, 0.01 MB. Copyright © 2023 Won et al. 2023 Won et al. https://creativecommons.org/licenses/by/4.0/ This content is distributed under the terms of the Creative Commons Attribution 4.0 International license.

### Live-cell confocal imaging.

Live-cell imaging of SMP1-1–GFP-expressing T. cruzi was performed as follows.

**(i) Amastigotes.** Approximately 2 × 10^6^ freshly isolated intracellular T. cruzi amastigotes in 150 μL of prewarmed imaging medium were placed directly on the glass coverslip of a 35-mm glass-bottom dish and allowed to settle at 37°C in a 5% CO_2_ incubator for 10 min. Seventy-five microliters of medium was removed prior to placing the dish on the microscope for imaging.

**(ii) Epimastigotes.** Approximately 2 × 10^5^ epimastigotes in 150 μL of LIT growth medium were placed directly on the glass coverslip of a 35-mm glass-bottom dish and allowed to settle prior to imaging. Parasites were imaged using a Yokogawa CSU-X1 spinning disk confocal system paired with a Nikon Ti-E inverted microscope and an iXon Ultra 888 EMCCD camera (100× objective). Image processing, analysis, and display were performed using ImageJ Fiji software (55).

### Indirect immunofluorescence microscopy.

Epimastigotes were fixed directly in growth medium with the addition of paraformaldehyde (1% final concentration in phosphate-buffered saline [PBS]) for 10 min at 4°C. Fixed parasites were pelleted by centrifuging for 10 min at 4,000 × *g* and resuspended in PBS. Ten microliters of the parasite solution was applied dropwise onto poly-l-lysine coated slides and allowed to dry completely prior to staining. For immunostaining of intracellular amastigotes, T. cruzi-infected NHDF on round cover glasses (12 mm; no. 1.5; Electron Microscopy Sciences, Hatfield, PA, USA) were fixed at 48 h postinfection with 1% (vol/vol) paraformaldehyde–PBS. All steps of the immunostaining protocol were preceded by three washes with PBS and carried out at room temperature. Parasites were permeabilized with a 0.1% Triton X-100 solution (JT Baker, Phillipsburg, NJ, USA) for 10 min and blocked with 3% bovine serum albumin (BSA) (Sigma-Aldrich, St. Louis, MO, USA) in PBS for 1 h. The primary antibody solution containing 1:400 mouse anti-FLAG (clone M2, Sigma-Aldrich, St. Louis, MO, USA) and/or 1:1,500 rabbit anti-FCaBP (56) in 1% BSA in PBS was added for 1 h, followed by a 1:1,000 anti-mouse Alexa Fluor 594 and/or anti-rabbit Alexa Fluor 647 solution in 1% BSA in PBS for 1 h. DAPI (4′,6-diamidino-2-phenylindole; 0.2 μg/mL; Thermo Fisher Scientific, Waltham, MA, USA) in PBS was added for 5 min, and following washes, coverslips were placed on slides with Prolong Diamond mountant (Thermo Fisher Scientific, Waltham, MA, USA) and imaged using a Yokogawa CSU-X1 spinning disk confocal system paired with a Nikon Ti-E inverted microscope and an iXon Ultra 888 EMCCD camera (100× objective). Image processing, analysis, and display were performed using ImageJ Fiji software (55).

### Detection of biotinylated protein fractions in T. cruzi.

**(i) Epimastigotes.** Epimastigotes (1.5 × 10^8^) were pelleted at 2,060 × *g* for 10 min, resuspended in 1 mL of LIT medium, and incubated with 50 μM biotin for 10 min at 37°C. Parasites were washed twice with ice-cold PBS and then resuspended in 1 mL cell lysis buffer (57) (0.5% Nonidet P-40, 500 mM NaCl, 5 mM EDTA, 1 mM dithiothreitol [DTT], 50 mM Tris base, 0.4% sodium dodecyl sulfate [SDS] [pH 7.4]) with Roche cOmplete protease inhibitor (Sigma-Aldrich, St. Louis, MO, USA), 100 μM PMSF, and 10 μM TLCK. Lysates were sonicated using 3 pulses of 30 s at 100% amplitude (Q700 sonicator; QSONICA, Newton, CT), with 15-s breaks between to cool the tubes on ice. Samples were centrifuged at 16,000 × *g* for 20 min at 4°C, and the supernatant was collected. Aliquots of clarified lysate (3 × 10^6^ parasite equivalents) were resolved by SDS-PAGE (Mini-PROTEAN TGX protein gel; Bio-Rad, Hercules, CA, USA), transferred to polyvinylidene difluoride (PVDF) membranes (Immobilon-FL; MilliporeSigma, Burlington, MA, USA), and probed with streptavidin-DyLight 800 (Thermo Fisher Scientific, Waltham, MA, USA) to detect biotinylated proteins.

**(ii) Intracellular amastigotes.** At 48 hpi, T. cruzi-infected NHDF monolayers in five T-150 flasks per parasite line were exposed to 100 μM biotin for 10 min at 37°C and 5% CO_2_. Monolayers were then rinsed three times with cold PBS and incubated with 2 mL of cell lysis buffer (as described above). Flasks were agitated manually for 5 min; then, cells were scraped and transferred into a tube containing 0.5 μL of Benzonase (Sigma-Aldrich, St. Louis, MO, USA). Tubes were placed on a rotative wheel for 15 min at room temperature and then sonicated as described above. Amastigote loading volumes were normalized via Western blotting as follows. Equal volumes of serially diluted protein lysates, generated for WT-, F-Turbo-, and C-Turbo-infected NHDF, were resolved by SDS-PAGE (Mini-PROTEAN TGX gels), transferred to PVDF membranes, and probed with a rabbit antibody to trypanosome Binding immunoglobulin protein (BiP) (1:5,000 dilution) (58), followed by anti-rabbit Alexa Fluor 647 (1:10,000 dilution) (for example, see Fig. S3). A LI-COR Odyssey CLx imager was used to measure BiP signal in each sample (Image Studio), and relative BiP signal densities were used to adjust the volumes of each amastigote lysate prior to loading onto streptavidin beads.

10.1128/msphere.00088-23.3 FIG S3 Western blot for normalization of amastigote protein across samples. Western blot where 4 serial dilutions of each sample’s protein lysate were loaded (in order), i.e., WT 1 to 4, F-Turbo 1 to 4, and C-Turbo 1 to 4, from the highest concentration to lowest. The blot was probed with anti-BiP, and a secondary antibody, anti-rabbit Alexa Fluor 647, was detected using fluorescence imaging. The relative BiP signals in each lane were used to calculate the volumes of each sample that would be loaded onto streptavidin-agarose beads as described in Materials and Methods. Download FIG S3, TIF file, 0.3 MB. Copyright © 2023 Won et al. 2023 Won et al. https://creativecommons.org/licenses/by/4.0/ This content is distributed under the terms of the Creative Commons Attribution 4.0 International license.

### Isolation of biotinylated proteins.

Protein lysates were loaded onto Pierce high-capacity streptavidin agarose (Thermo Fisher Scientific, Waltham, MA, USA). Packed-bead volumes of 100 μL and 150 μL were used for epimastigote and amastigote samples, respectively, and samples were incubated on a rotative wheel overnight at 4°C. For all following steps, washes consisted of adding 1 mL of the indicated buffer, placing the tube on the rotative wheel for 5 min, and then spinning down the agarose beads for 1 min at 500 × *g*, as previously described (57). Beads with bound protein were subjected to 5 washes with buffer 1 (8 M urea, 200 mM NaCl, 100 mM Tris [pH 8.0]) with 0.2% SDS, 5 washes with buffer 1 containing 2% SDS, and 5 washes with buffer 1 with no SDS at room temperature. Next, 2 washes with 200 mM NaCl–100 mM Tris at pH 7.0 and 2 washes with Tris at pH 8.0 were completed at 4°C. Washed beads were adjusted to pH 7.5 with 200 mM HEPES, and bound proteins were reduced using 5 mM dithiothreitol (Sigma-Aldrich) at 37°C for 1 h, followed by alkylation of cysteine residues using 15 mM iodoacetamide (Sigma-Aldrich) in the dark at room temperature for 1 h. Excessive iodoacetamide was quenched using 10 mM dithiothreitol. Protein mixtures were diluted 1:6 (vol/vol) using ultrapure water prior to digestion using sequencing-grade trypsin (Promega) at 37°C for 16 h. Digested peptides were subsequently desalted using self-packed C_18_ StageTips (3M Empore) (59) for LC-MS/MS analysis.

### Mass spectrometry.

**(i) Epimastigotes.** Desalted peptides were resuspended in 0.1% (vol/vol) formic acid and loaded onto HPLC-MS/MS system for analysis on an Orbitrap Q-Exactive Exploris 480 (Thermo Fisher Scientific) mass spectrometer coupled to an Easy nanoLC 1000 (Thermo Fisher Scientific) with a flow rate of 250 nL/min. The stationary-phase buffer was 0.5% formic acid, and the mobile phase buffer was 0.5% (vol/vol) formic acid in acetonitrile. Chromatography for peptide separation was performed using an increasing organic proportion of acetonitrile (5 to 40% [vol/vol]) over a 120-min gradient) on a self-packed analytical column using a PicoTip emitter (New Objective, Woburn, MA) using Reprosil Gold 120 C_18_, 1.9-μm-particle-size resin (Dr. Maisch, Ammerbuch-Entringen, Germany). The mass spectrometry analyzer operated in data-dependent acquisition mode with a top 10 method at a mass range of 300 to 2,000 Da. Data were processed using MaxQuant software (version 1.5.2.8) (60) with the following settings: oxidized methionine residues and protein N-terminal acetylation as variable modifications; cysteine carbamidomethylation as a fixed modification; first search peptide tolerance, 20 ppm; main search peptide tolerance, 4.5 ppm. Protease specificity was set to trypsin with up to 2 missed cleavages allowed. Only peptides longer than five amino acids were analyzed, and the minimal ratio count to quantify a protein was 2 (proteome only). The false discovery rate (FDR) was set to 1% for peptide and protein identifications. Database searches were performed using the Andromeda search engine integrated into the MaxQuant environment (61) against the UniProt Trypanosoma cruzi strain CL Brener (352153) database containing 19,242 entries (March 2020). The “matching between runs” algorithm with a time window of 0.7 min was employed to transfer identifications between samples processed using the same nanospray conditions. Protein tables were filtered to eliminate identifications from the reverse database and common contaminants. Filtered protein intensity scores are listed as “0.”

**(ii) Amastigotes.** Desalted peptides were resolubilized in 0.1% (vol/vol) formic acid and loaded onto an HPLC-MS/MS system for analysis on an Orbitrap Q-Exactive Exploris 480 (Thermo Fisher Scientific) mass spectrometer coupled to an FAIMS Pro Interface system and Easy nanoLC 1000 (Thermo Fisher Scientific) with a flow rate of 300 nL/min. The stationary-phase buffer was 0.1% formic acid, and the mobile phase buffer was 0.1% (vol/vol) formic acid in 80% (vol/vol) acetonitrile. Chromatography for peptide separation was performed using increasing organic proportion of acetonitrile (5 to 40% [vol/vol]) over a 120-min gradient) on a self-packed analytical column using a PicoTip emitter (New Objective, Woburn, MA) using Reprosil Gold 120 C_18_, 1.9-μm-particle-size resin (Dr. Maisch, Ammerbuch-Entringen, Germany). High-precision indexed retention time (iRT) calibration was used for samples processed using the same nanospray conditions (62). The mass spectrometry analyzer operated in data-independent acquisition mode at a mass range of 300 to 2,000 Da and compensation voltages (CVs) of −50/−70 CVs with survey scans at 120,000 and 15,000 resolutions at the MS1 and MS2 levels, respectively. Data were processed using Spectronaut software (version 15; Biognosys AG) (63) using directDIA analysis with default settings, including oxidized methionine residues, biotinylation, and protein N-terminal acetylation as variable modifications, cysteine carbamidomethylation as a fixed modification, and an initial mass tolerance of MS1 and MS2 of 15 ppm. Protease specificity was set to trypsin, with up to 2 missed cleavages allowed. Only peptides longer than seven amino acids were analyzed, and the minimal ratio count to quantify a protein was 2 (proteome only). The FDR was set to 1% for peptide and protein identifications.

Database searches were performed against the UniProt Trypanosoma cruzi strain CL Brener (352153) database containing 19,242 entries (March 2020). Protein tables were filtered to eliminate identifications from the reverse database and common contaminants. Filtered protein intensity scores are listed as “filtered.”

### Principal-component analysis.

All protein intensity scores were uploaded to Metaboanalyst 5.0 (64) to perform statistical analysis, with one factor. Data was entered as peak intensities, filtered using the interquartile range, and then normalized by sum and log transformed. Two-dimensional PCA scores were plotted in Prism GraphPad.

### Volcano plots.

For epimastigote data, protein intensity scores for all proteins that were found to be enriched 100-fold or more in the F-Turbo or C-Turbo samples over the wild type (data from Table S2) were entered into Prism GraphPad and log_10_ transformed. Multiple unpaired *t* tests were run on the data with a false discovery rate of 1%, and the results were reported as (F-Turbo score) − (C-Turbo score), with −log_10_(*q* value) reported for the volcano plot. For amastigote data, Spectronaut software was used to generate the statistical analysis of the amastigote proteomics (data from Table S1). Within the data-independent acquisition (DIA) analysis pipeline, the default settings were used, including a false discovery rate of 1%, and an unpaired Student’s *t* test was performed. Fold changes were reported as an average log_10_(epimastigotes) or log_2_(amastigotes) ratio for the volcano plot. The volcano plot was created in Prism GraphPad. Postanalysis, six proteins were excluded from the list of those enriched in amastigote flagella, as they were not identified in 2 of 3 biological replicates. Additionally, all allelic duplicates were removed to ensure that the proteins listed in the final tables were represented by a single gene identifier corresponding to the CL Brener reference genome (TriTrypDB [https://tritrypdb.org/]), but paralogs remain.

### Interpro domain enrichment analysis.

DAVID (65) enrichment was used to assign Interpro domains to all of the proteins found to be significantly enriched in either of the F-Turbo samples. No weighting of the data was completed. Interpro domains with a *P* value of ≤0.05 were considered significantly enriched.

### Ortholog identification.

TriTrypDB was used to identify potential orthologs of the proteins we identified through proximity labeling. The TriTrypDB IDs listed in Table S3 were used to search for orthologs in either T. brucei or Leishmania mexicana based on primary amino acid sequence.
