# Supplementary figures and images for: Proximity-Dependent Biotinylation and Identification of Flagellar Proteins in Trypanosoma cruzi
Source: mSphere. 2023 Apr 5;8(3):e00088-23. doi: 10.1128/msphere.00088-23 (PMC10286712; doi:10.1128/msphere.00088-23)

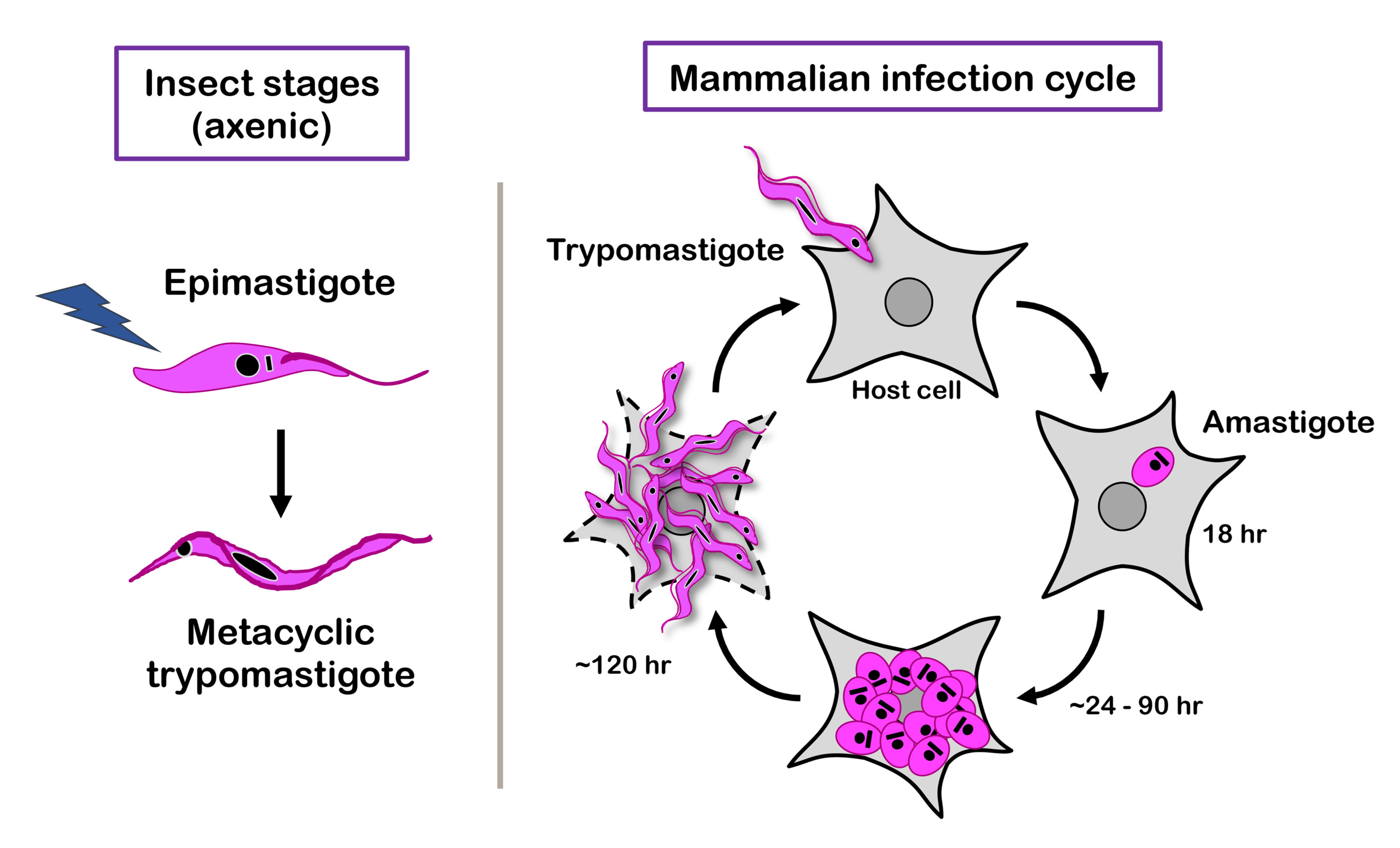

Supplement: FIG S1 [file msphere.00088-23-s0001.tif]

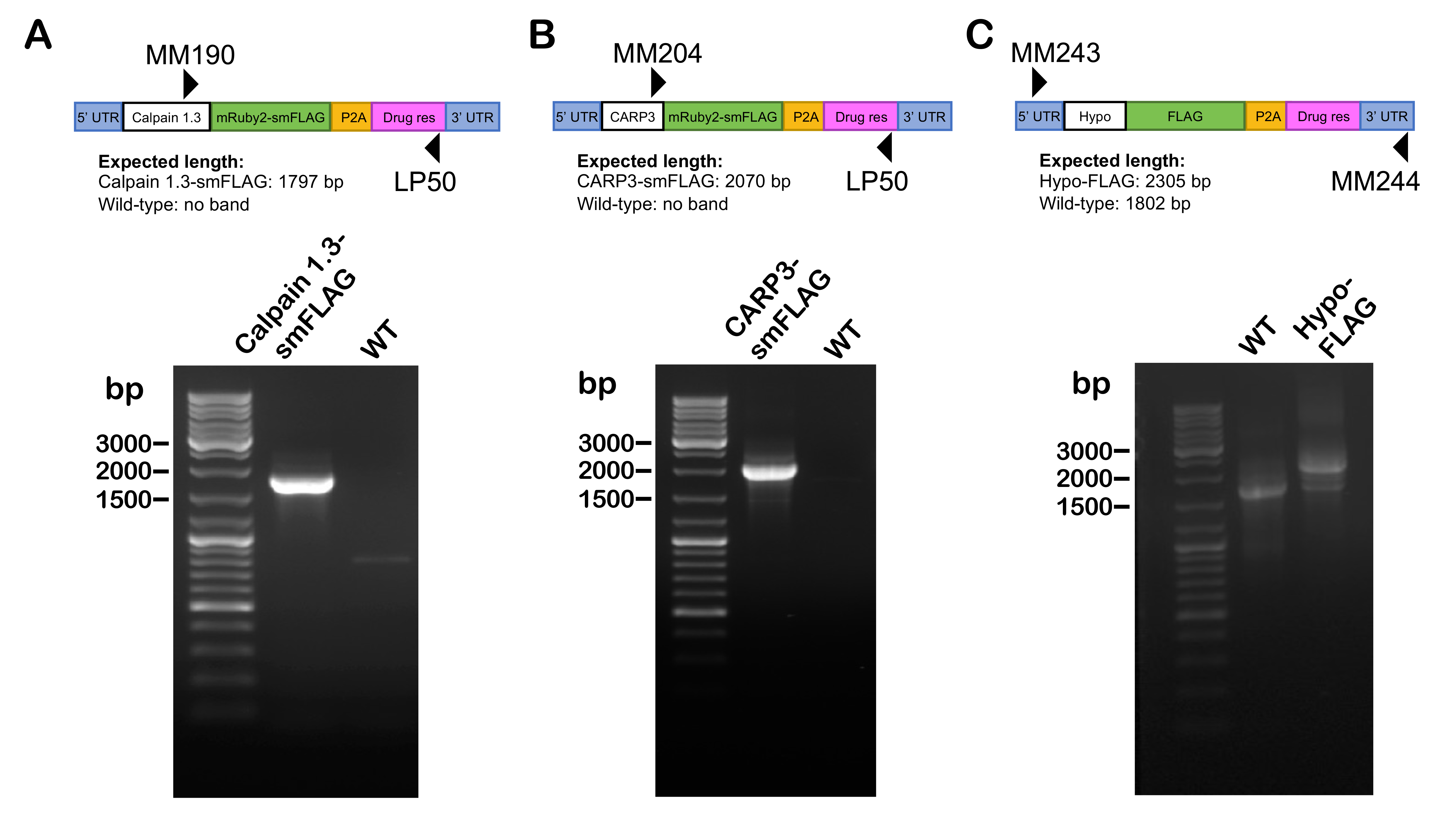

Supplement: FIG S2 [file msphere.00088-23-s0002.tif]
